# Enzymatic and Algebraic Methodology to Determine the Contents of Kunitz and Bowman–Birk Inhibitors and Their Contributions to Total Trypsin or Chymotrypsin Inhibition in Soybeans

**DOI:** 10.1021/acs.jafc.3c06389

**Published:** 2024-05-08

**Authors:** Keshun Liu

**Affiliations:** Grain Chemistry and Utilization Laboratory, National Small Grains and Potato Germplasm Research Unit, U.S. Department of Agriculture, Agricultural Research Service, 1691 S. 2700 W, Aberdeen, Idaho 83210, United States

**Keywords:** method, soybeans (*Glycine max*), trypsin inhibitors, chymotrypsin inhibitors, Kunitz inhibitor, Bowman–Birk inhibitor, protease inhibitors, analysis

## Abstract

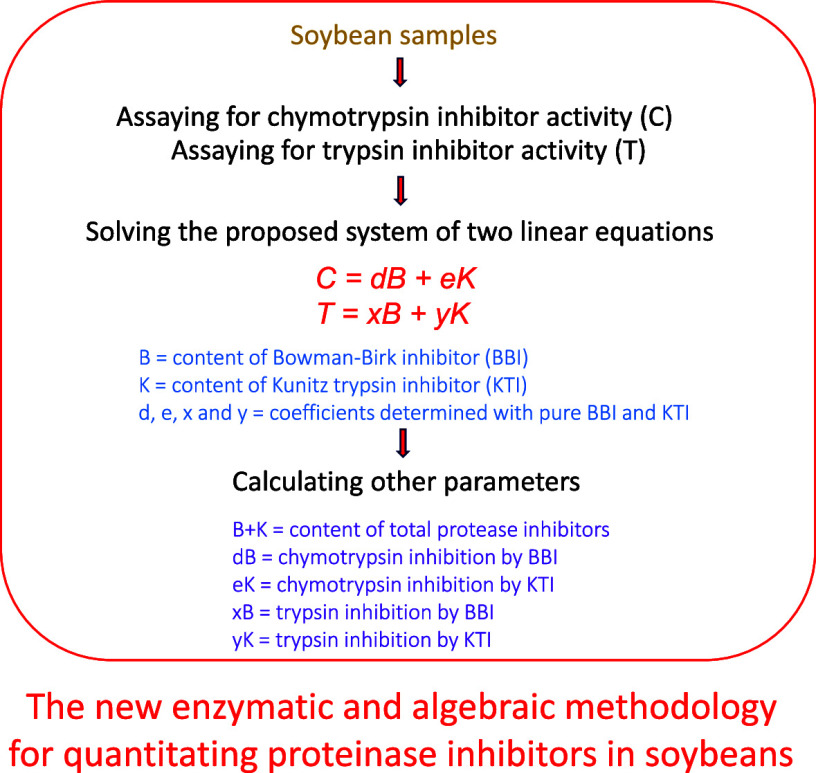

Soybeans are the number one source of plant proteins for food and feed, but the natural presence of protein protease inhibitors (PIs), namely, the Kunitz trypsin inhibitor (KTI) and the Bowman–Birk inhibitor (BBI), exerts antinutritional effects. This communication describes a new methodology for simultaneously quantitating all parameters of PIs in soybeans. It consists of seven steps and featured enzymatically measuring trypsin and chymotrypsin inhibitory activities, respectively, and subsequently determining the contents of reactive KTI and BBI and the contributions of each toward total PI mass and total trypsin or chymotrypsin inhibition by solving a proposed system of linear equations with two variables (*C* = *dB* + *eK* and *T* = *xB* + *yK*). This enzymatic and algebraic (EA) methodology was based on differential inhibitions of KTI and BBI toward trypsin and chymotrypsin and validated by applications to a series of mixtures of purified KTI and BBI, two KTI-null and two conventional soybeans, and by sodium dodecyl sulfate polyacrylamide gel electrophoresis. The EA methodology allowed calculations of PI composition and the contributions of individual inhibitors toward total inhibition with ease. It was first found that although BBI constituted only about 30% of the total PI mass in conventional raw soybeans, it contributed about 80% toward total chymotrypsin inhibitor activity and about 45% toward trypsin inhibitor activity. Therefore, BBI caused more total protease inhibitions than those of KTI. Furthermore, the so-called KTI-null soybean mutants still contained measurable KTI content and thus should be named KTI-low soybeans.

## 1 Introduction

Soybeans (*Glycine max*) contain about 40% protein and 20% oil and have been the most valuable legumes and oilseeds for food and feed. Yet, like all other legume seeds, soybeans contain protease inhibitors (PIs) of protein nature, which are considered antinutritional.^1,2^ In soybeans, there are two types of PI: the Kunitz trypsin inhibitor (KTI) and the Bowman–Birk inhibitor (BBI). Soybean KTI was first isolated and crystallized by Kunitz.^3^ It consists of 181 amino acids, with the reactive site being located at Arg 63 and Ile 64 and has a molecular weight (MW) of about 20.1 kDa with two disulfide bonds.^4^ Soybean BBI was first isolated by Bowman^5^ and then purified by Birk.^6^ It consists of 71 amino acids cross-linked by 7 disulfide bonds, with a MW of about 8 kDa and has two independent binding sites: a trypsin-reactive site (Lys 16 and Ser 17) and a chymotrypsin-reactive site (Leu 43 and Ser 44).^7^

Because of the difference in structure, the two PIs in soybeans exhibit significant differences in their genetic constitutions,^8−11^ biological and physicochemical properties,^1,3^ and nutritional and health implications.^2,12^ KTI has inhibition primarily toward trypsin and slightly toward chymotrypsin.^3^ In contrast, BBI inhibits trypsin and chymotrypsin independently and at about equal capacities.^1,7^ Although both KTI and BBI are considered antinutritional due to their interference with protein digestion and implication for pancreatic enlargement,^1,2,12^ BBI has been found beneficial to humans and animals by exerting therapeutic effects.^12−14^

There are two parameters of interest when we describe PIs in soybeans and other proteinaceous materials. One is inhibitor activity, and the second is the inhibitor concentration or content. Inhibitor activity refers to the activity of a reactive inhibitor toward a protease for a given amount of sample, while the inhibitor concentration is the absolute amount of a reactive inhibitor per unit sample. In general, it is the content of a reactive inhibitor that determines its inhibitory activity toward a protease. Because PI in soybeans could have adverse^2^ and/or beneficial^13^ effects, it would be of great significance if we can differentiate between the two inhibitors, with respect to their contents and activities, and then calculate the contribution of each inhibitor toward total PI mass (also known as PI composition), total trypsin inhibition, and total chymotrypsin inhibition for a given sample amount.

Historically, trypsin inhibitor activity (TIA) in soybeans and soy products has been of primary interest for measurement using various enzymatic methods. Although the methodology, which is based on measuring the difference in absorbance between the absence and presence of an inhibitor, has gone through significant improvements for accuracy, simplicity, and standardization,^15−18^ it does not differentiate KTI from BBI in any way because both inhibitors inhibit trypsin. Consequently, over the years, several nonenzymatic methods have been developed or used to measure the content of an individual inhibitor (KTI or BBI). In general, they fall into three major categories: immunoassays, polyacrylamide gel electrophoresis (PAGE), and liquid chromatography. Immunoassays are exemplified by the enzyme-linked immunosorbent assay (ELISA) using polyclonal or monoclonal antibodies to KTI or BBI,^19,20^ while PAGE, either native^21,22^ or sodium dodecyl sulfate (SDS),^23^ requires coupling with densitometers or other gel imaging technology. Chromatographic quantitation was originally limited to KTI^24,25^ and has lately expanded to include BBI as well.^26^ As with most analytical methods, these methods and their variants have pros and cons. Oftentimes, two or more methods are combined for achieving a research objective, leading to increased complexity and cost.^10,22^

There has been no single method that can measure both types of inhibitors, not only for their mass composition but also for their contribution toward total trypsin inhibition and total chymotrypsin inhibition. A new methodology developed in the present study and reported herein has such a capacity. It is enzymatic and is based on a proposed system of linear equations with two variables. The methodology can be very useful for researchers to determine the levels of both KTI and BBI simultaneously in soybeans, facilitate their efforts to modify inhibitor content and activity through genetic manipulation and/or processing, and thus achieve optimal nutrition and health benefits of soybeans as food and feed.

## 2 Materials and Methods

All chemicals were purchased from Sigma-Aldrich, Inc. (St. Louis, MO, USA). They included (1) crystalline bovine trypsin (T4126, essentially salt-free, lyophilized powder), (2) α-chymotrypsin from bovine pancreas (C4129, lyophilized powder), (3) *N*α-benzoyl-dl-arginine-*p*-nitroanilide (dl-BAPA) (B4875), (4) *N*-benzoyl-l-tyrosine-*p*-nitroanilide (BTNA) (B6760), (5) purified soybean KTI (T2327, mass purity, 96%), (6) purified soybean BBI (T9777, mass purity, 88%), (7) dimethyl sulfoxide, (8) dimethylformamide (DMF), (9) Tris base (hydroxymethyl) amino-methane, and (10) calcium chloride dihydrate. All chemicals were of ACS grade, except for enzyme and inhibitor preparations. Deionized water was used throughout the study.

Three accessions of raw soybeans, including two genotypes lacking KTI and one normal cultivar (wild type), were kindly provided by the U.S. Department of Agriculture, Agricultural Research Service, Soybean Germplasm Collection, Urbana, IL 61801, USA, upon request. The fourth sample of raw soybeans was purchased locally. Although its variety was unknown, it served as another wild type.

### 2.1 Experimental Design

For method development and validation, three experiments were carried out and each involved measurement of TIA and chymotrypsin inhibitor activity (CIA) for relevant samples. In experiment 1, purified KTI and BBI were measured in triplicate. The purpose was to determine the four coefficients for a proposed system of linear equations with two variables. Stock solutions (200 μg/mL, adjusted to 100% pure) of KTI and BBI reagents were prepared using water. Working solutions (15 mL) of pure BBI and KTI were made from the BBI and KTI stock solutions, respectively, by diluting each with water so that 1 mL caused 50–60% trypsin or chymotrypsin inhibition.

In experiment 2, a series of 20% incremental mixtures of purified KTI and BBI were made and measured for TIA and CIA in duplicate. The objective was to validate the new methodology by comparing the estimated % KTI in the mixtures with the actual % KTI. Stock solutions of incremental mixtures were made from the pure KTI and BBI stock solutions (prepared in experiment 1) by incremental combinations of 80/20% KTI/BBI, 60/40% KTI/BBI, 40/60% KTI/BBI, and 20/80% KTI/BBI. For example, 16 mL of pure KTI stock solution was mixed with 4 mL of pure BBI stock solution to create 20 mL of a new stock solution containing 80% KTI and 20% BBI. The total PI concentration in each incremental stock solution remained at 200 μg/mL. Incremental working solutions were made from these incremental stock solutions in the same way as making the working solutions of pure KTI and BBI in expt. 1.

In experiment 3, the four raw soybean samples, featuring two KTI-null and two wild types, were measured in duplicate. This was followed by nonreducing tricine SDS-PAGE for protein extracts. The objective was to further validate the new methodology by comparing the estimated amounts of KTI and BBI in real soybeans (mass composition) and other parameters with those reported by previous investigators.

### 2.2 Extracting Inhibitors from Soybeans

All soybean samples were ground to pass through a U.S. standard no. 50 mesh (equivalent to a 300 μm opening). For extracting PI (both KTI and BBI) from soybeans, 0.8 g of ground sample was extracted with 40 mL of 10 mM NaOH and magnetic stirring at 500 rpm for 3 h. For the TIA assay, the sample suspension was diluted with deionized water so that 1 mL of a dilute sample caused 30–70% trypsin inhibition after correcting for blank readings. For the CIA assay, the sample suspension was diluted with deionized water so that 1 mL of a dilute sample caused 40–60% chymotrypsin inhibition after correcting for blank readings.

### 2.3 Trypsin Inhibitor Activity Assay

TIA in all samples (purified PI or raw soybeans) was measured by AOCS Ba 12a-2020,^18^ which was mainly based on the 5 mL method reported elsewhere.^16,17^ In brief, after proper working solutions of purified KTI and BBI were made or after soybean samples were ground, extracted, and properly diluted as described above, the colorimetric assay was conducted in a water bath at 37 °C and initiated by mixing 1 mL of a dilute extract (or a working solution of purified KTI and/or BBI) mixed with 2.5 mL of DL-BAPA working solution in a test tube. This was followed by adding 1 mL of trypsin working solution to start the colorimetric reaction. Exactly 10 min later, 0.5 mL of 30% acetic acid was added to stop the reaction. The reaction mixture (a total of 5 mL) was centrifuged and measured for absorbance at 410 nm as a sample reading. For a reference reading, 1.0 mL of water was used instead of the dilute extract. The tests for sample and reference readings were duplicated. A sample blank and a reference blank were also prepared and measured.

One trypsin unit (TU) is defined as an increase of 0.02 absorbance at 410 nm under the 5 mL assay condition specified in the method. TIA is expressed in two units: the arbitrary unit as trypsin units inhibited (TUI) per mg sample and the absolute amount (μg) of trypsin inhibited (TId) per mg sample (same as mg of TId/g sample). The relationship between the two units was based on a standardized conversion factor of 1 μg trypsin = 1.5 TU (aka 1 μg trypsin = 0.03 A410), whereas trypsin has a specific activity of 15,000 BAEE (*N*α-benzoyl-l-arginine ethyl ester) units/mg protein, equivalent to 150 BAPA units/mg protein, according to Liu.^17^ Such a relationship can be simply described as mg TId/g sample = (TUI/mg sample)/1.5.

### 2.4 Chymotrypsin Inhibitor Activity Assay

For the CIA assay, the method of Liu^27^ was used. The extraction for the CIA assay was the same as that for the TIA assay (see description above). The colorimetric assay procedure for CIA^27^ was like the assay procedure for TIA^16,17^ with respect to reaction time, temperature, and total assay volume. However, the CIA assay differed in several aspects from the TIA assay. These included (1) the extent of diluting a sample extract, (2) the use of BTNA as a substrate, (3) the presence of the organic solvent, DMF, (4) the use of serial levels of a dilute extract, (5) measurement of absorbance at 400 nm, and (6) the definition and equations used for calculating inhibitor activities.

Regarding the last item, one chymotrypsin unit (CU) is defined as an increase of 0.01 absorbance at 400 nm under the conditions of the assay method of Liu.^27^ With this definition, CIA is also expressed in two units: the arbitrary unit as chymotrypsin units inhibited (CUI) per mg sample and the absolute amount (μg) of chymotrypsin inhibited (CId) per mg sample (same as mg CId/g sample). The relationship between the two units was based on a standardized conversion factor of 1 μg chymotrypsin = 1.5 CU, whereas chymotrypsin has a specific activity of 50 BTEE (*N*-benzoyl-l-tyrosine ethyl ester) units/mg protein, equivalent to 150 BTNA units/mg protein, according to Liu.^28^ Such a relationship can be simply described as mg CId/g sample = (CUI/mg sample)/1.5.

### 2.5 Tricine SDS-PAGE

Nonreducing tricine SDS-PAGE was performed for extracts of four soybean accessions (two KTI-nulls and two wild types) to identify any differences in PI composition based on band intensity against the two purified inhibitors (KTI and BBI). The procedure described by Schägger^29^ was adapted with some modifications. In brief, a precast 16.5% Tris–tricine gel fitted for a Criterion cell (a midi-format system) from Bio-Rad (Hercules, CA, USA) was used. The protein extract (used for PI assays) for each accession was clarified, measured for protein content with a Coomassie protein assay kit (Fisher Scientific, Waltham, MA, USA), and diluted to 0.75 mg of protein/mL. After this step, 0.15 mL of each diluted extract was mixed with 0.15 mL of tricine SDS sample buffer (without a reducing agent). The same treatment was applied to each stock solution of purified inhibitors (KTI and BBI). After the mixture was heated at 95 °C for 7 min, 15 μL of each cooled solution was loaded onto the gel, along with a solution of protein markers having a range of MWs (also from Bio-Rad). The gel was run at 125 V (with the maximum current limit set at 100 mA) for 2 h and 20 min. At the end of electrophoresis, the gel was fixed, stained with G-250, and destained until most bands were resolved from the background.

## 3 Results and Discussion

For the past few years at our USDA laboratory, two enzymatic methods have been significantly improved and optimized to measure TIA^16^ and CIA^27^ in soybeans and other protein products, respectively, with good sensitivity and accuracy. Furthermore, with the two new methods, inhibitory activities can now be expressed in either arbitrary units or absolute amounts of enzyme inhibited upon standardization against reference trypsin or chymotrypsin with known specific activity.^17,28^ The method for the TIA assay has been approved as the AOCS Official Method Ba-20a-2020.^18^ The new methodology to determine proteinase inhibitor composition and contributions to total inhibition, as reported in this article, was developed out of the two improved methods of assaying TIA and CIA in soybeans, followed by a novel algebraic treatment. Therefore, it is termed the enzymatic and algebraic methodology or simply the EA methodology.

### 3.1 Proposed System of Linear Equations with Two Variables: A Theoretical Basis for Developing the EA Methodology

It has been known that KTI and BBI are two major types of proteinase inhibitors in soybeans and that BBI has almost equal inhibitory actions toward trypsin and chymotrypsin,^1,6^ while KTI has a strong inhibition toward trypsin but a weak inhibition toward chymotrypsin.^3^ Based on this principle, a system of linear equations with two variables is proposed, as shown in Table 1. The two variables, *B* and *K*, in eqs 1 and 2 represent concentrations of BBI and KTI in a test soybean sample, respectively. In eq 1, *C* represents the CIA of the sample and is a sum of CIA by KTI and CIA by BBI. Similarly, *T* in eq 2 represents the TIA of the sample and is a sum of TIA by KTI and TIA by BBI. Both *C* and *T* are functions of *B* and *K*. Each linear equation has two coefficients: *d* and *e* are associated with *C*, whereas *x* and *y* are associated with *T*. By solving the proposed system of linear eqs 1 and 2, the two variables, *B* and *K*, can be determined using two of the four derived general equations (eqs 3–6). Among the four, eq 3 requires prior determination of *K*, while eq 5 requires prior determination of *B*. However, eqs 4 and 6 are independent and do not require a prior determination of *K* or *B*. Therefore, eqs 4 and 6 are preferred, even though they appear more complex than eqs 3 and 5.

**Table 1:** Proposed System of Linear Equations with Two Variables to Estimate PI Composition in Soybeans

explanations	equations (no.)
two general interrelated linear equations with two variables	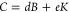 1
	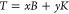 2
*C* and *T* represent CIA and TIA, respectively, measured for a test sample^a^^,^^b^^,^^c^	
*B* and *K* are the two variables to be determined, representing the concentrations of BBI and KTI in the test sample, respectively^d^	
*d* and *e* are coefficients, representing CIA (*C* values) of 100% pure BBI and KTI, respectively^e^^,^^g^	
*x* and *y* are coefficients, representing TIA (*T* values) of 100% pure BBI and KTI, respectively^f^^,^^g^	
option 1: to determine the *K* value first	
first, from eq 1, find *B* in relation to *K*	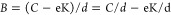 3
then, substitute eq 3 into 2 to determine *K*	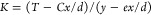 4
option 2, to determine the *B* value first	
first, from eq 1, find *K* in relation to *B*	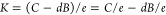 5
then, substitute eq 5 into 2 to determine *B*	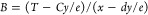 6
concentration of total PIs (*P*) in μg/mg sample (or mg/g)	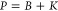 7
% KTI relative to the total mass in the sample	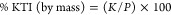 8
% KTI relative to the total CIA in the sample	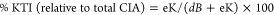 9
% KTI relative to total TIA in the sample	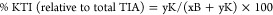 10
% BBI (relative to total mass, TIA or CIA)	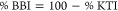 11

a CIA: chymotrypsin inhibitor activity; TIA: trypsin inhibitor activity.

b The CIA unit can be CUI or μg CId per mg (soy samples) or per μg (purified BBI or KTI).

c The TIA unit can be TUI or μg TId per mg (soy samples) or per μg (purified BBI or KTI).

d The unit for *B* and *K* is mg pure inhibitor/g sample (same as μg/mg).

e *d* = *C* of 100% pure BBI; *e* = *C* of 100% pure KTI.

f *x* = *T* of 100% pure BBI; *y* = *T* of 100% pure KTI.

g Each type of the units (arbitrary or absolute amounts), once selected, need to be consistent for purified BBI, purified KTI, and test samples.

Once *K* and *B* are determined from eqs 3–6, the concentration of total PI (*P*) in the soy sample can be determined by using eq 7, while % KTI to total PI mass (i.e., PI composition) can be determined using eq 8. Furthermore, % KTI relative to total CIA and total TIA in the sample can be easily determined using eqs 9 and 10, respectively. Once % KTI is determined, % BBI relative to total mass, total CIA, or TIA can be easily determined with eq 11.

### 3.2 Seven Steps of the EA Methodology

**Figure 1:**
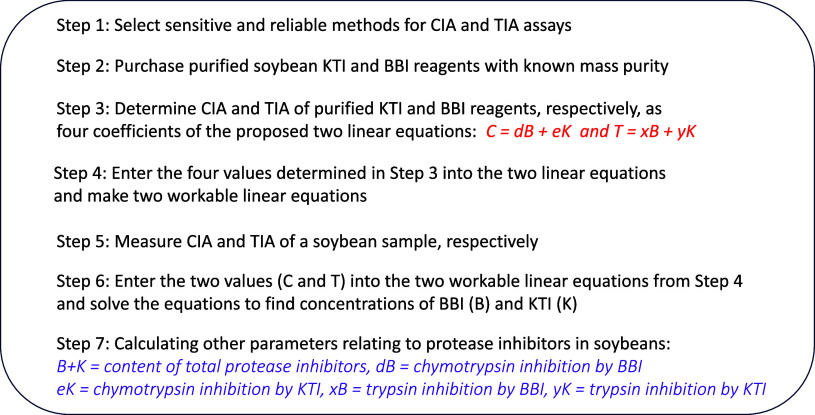
Flowchart of the seven-step methodology for determining soybean PI composition and related parameters.

With the proposed system and general equations provided in Table 1, in developing the new methodology for estimating several important parameters with regard to the PI composition and contribution of each PI (BBI or KTI) to total CIA and TIA in soybeans, the following key steps need to be conducted. Figure 1 provides a schematic flowchart of the EA methodology.

1 Choose sensitive and reliable methods for measuring TIA and CIA in soybeans and use them as the basis for developing the new methodology. Since it was based on the measurement of the total TIA and CIA of soybeans, the new methodology requires sensitive and accurate methods for measuring both types of inhibitory activities. Fortunately, recent progress in method development at our USDA laboratory has made it possible to meet this requirement. Specifically, for the present study, the AOCS official method Ba 12a-2020^16−18^ was used for the TIA assay, while the method of Liu^27,28^ was used for the CIA assay. Both have recently been significantly improved and optimized based on prior methods.
2 Purchase purified soybean KTI and BBI reagents with known protein content (also known as mass purity, the higher, the better). By measuring purified KTI and BBI for CIA and TIA, the four coefficients, *d*, *e*, *x*, and *y*, can be determined. For the present study, purified KTI and BBI reagents were both purchased from Sigma-Aldrich, with a mass purity of 96 and 88%, respectively.
3 Determine *C* and *T* of the two purified inhibitors, respectively, using the two improved methods for assaying CIA^27^ and TIA.^16,18^ By using the two methods, CIA and TIA can be expressed in two units: the arbitrary units as CUI/mg sample and TUI/mg sample, respectively, and the absolute amounts of protease inhibited as μg CId/mg sample^28^ and μg TId/mg sample,^17^ respectively. Therefore, two corresponding sets (A and B) of four coefficients were determined, as shown in Table 2.
4 Enter the four coefficients determined from step 2 (Table 2) into eqs 1 and 2 in Table 1. This generates workable equations for finding *B* and *K*. For the present study, since two sets of coefficients were determined by using two sets of inhibitory units (Table 2), two corresponding sets (A and B) of workable equations, based on the general equations in Table 1, were developed and shown in Table 3.
5. Determine the test sample(s) for *C* and *T* values using the same methods for measuring purified KTI and BBI reagents. In the present study, for demonstrating how the new methodology works and for validating its performance in estimating PI composition, two groups of the test samples were used. The first group consisted of the four solutions containing purified KTI and BBI reagents with KTI at 20, 40, 60, and 80% (w/w), the 100% pure BBI (0% KTI), and the 100% pure KTI solution. The second group included two KTI-null and two normal soybean varieties. The results are presented and discussed in the following sections.
6. Enter the *C* and *T* values into workable eqs 1 and 2 in Table 3 and find *B* and *K* by solving the system of linear equations with two variables using two of the four workable eqs 3–6 shown in Table 3. Since two different units were used to express CIA and TIA of purified inhibitors (expt. 1) in the present study, for determining PI composition in a series of mixtures of the two purified inhibitors (expt. 2) and four soybean samples (expt. 3), either one of the two sets (A and B) of workable equations shown in Table 3 could be used to find *B* and *K* values. However, the inhibition units, once selected to express CIA and TIA, must be consistent for purified inhibitors and test samples. Specifically, if CIA and TIA measured in test samples are expressed in the arbitrary units, the A set of equations needs to be used. On the other hand, if CIA and TIA measured in test samples are expressed in the absolute amounts of protease inhibited, the B set of equations needs to be used. Furthermore, in using either A (eqs 3a–6a) or B (eqs 3b–6b) set (Table 3), all formulas (initial, intermedium, or final, as the simplifying step progressed) in each equation should lead to the same *K* and *B* values.
7. With *K* and *B* values determined from the above steps, use the general eqs 7 to 11 in Table 1 to determine the total PI content, % KTI by mass or % BBI by mass (PI composition), % KTI contribution to total CIA, % KTI to total TIA, % BBI to total CIA, and % KTI to total CIA, respectively.

**Table 2:** Coefficients for the Proposed System of Two Linear Equations with Two Variables, Determined by Measuring the CIA and TIA of Purified Bowman-Birk Inhibitor and Kunitz Inhibitors from Soybeans, Adjusted to 100% Pure^a^

	chymotrypsin inhibitor activity (CIA)^b^		trypsin inhibitor activity (TIA)^c^	
A set: based on arbitary units	coefficients^d^	CUI/μg	coefficients^d^	TUI/μg
pure (100%) Bowman–Birk inhibitor	*d*	3.78 ± 0.06	*x*	3.32 ± 0.04
pure (100%) Kunitz trypsin inhibitor	*e*	0.37 ± 0.01	*y*	1.68 ± 0.04
B set: based on amounts of protease inhibited	coefficients^d^	μg CId/μg	coefficients^d^	μg TId/μg
pure (100%) Bowman–Birk inhibitor	*d*	2.52 ± 0.04	*x*	2.21 ± 0.03
pure (100%) Kunitz trypsin inhibitor	*e*	0.25 ± 0.01	*y*	1.12 ± 0.03

a Mean of triplicate measurements ± standard deviation.

b CIA can be expressed in CUI, an arbitrary unit or amounts of CId.

c TIA can be expressed in TUI, an arbitrary unit or amounts of TId.

d For the definition of each coefficient, refer to Table 1 for the proposed model.

Although CUI and TUI are both arbitrary units, they have totally different definitions under two different assay systems. Therefore, CIA values in the CUI and TIA values in TUI cannot be simply summed. Similarly, both CId and TId feature absolute amounts of protease inhibited, but they have different meanings under two different assay systems and are standardized against different proteases.^17,28^ Therefore, the values of the two cannot be simply totaled either. Furthermore, the standardized conversion factor for CUI to CId, determined under the assay conditions of Liu,^27^ is 1.5,^28^ which happens to be the same as that between TUI and TId,^17^ determined under the assay conditions of Liu.^16^ Therefore, the four coefficients in the B set are the quotients of the corresponding coefficients in the A set divided by 1.5.

Compared with KTI, BBI had about 10 times higher inhibition toward chymotrypsin and about 2 times higher inhibition toward trypsin (Table 2). During the time when both KTI and BBI were first discovered and isolated, it was already known that KTI has a strong inhibition toward trypsin but a weak inhibition toward chymotrypsin,^3^ while BBI has about equal inhibition toward both enzymes.^1,6^ The present study (Table 2) not only confirmed the pioneers’ observations but also was the first to provide numerical values about the differences in inhibitions toward trypsin and chymotrypsin by KTI and BBI, respectively. The measured difference in trypsin inhibition based on the mass unit between BBI and KTI can be attributed to the difference in the MW of the two inhibitors, while the sharp difference in chymotrypsin inhibition can be explained by the fact that BBI has two independent reactive sites (one for trypsin and the other for chymotrypsin), while KTI only has one reactive site.^3−7^

**Table 3:** Linear Equations with Coefficients Determined for Estimating PI Composition in Soybeans

general equations^a^	equations with units of CUI and TUI^b^^,^^d^	equations with units of CId and TId^c^^,^^d^
two general interrelated linear equations		
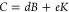	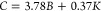 1a	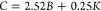 1b
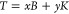	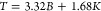 2a	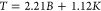 2b
option 1: to determine the *K* value first		
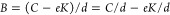	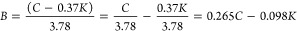 3a	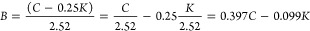 3b
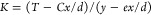	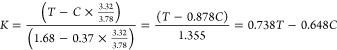 4a	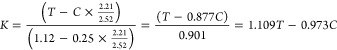 4b
option 2, to determine the *B* value first		
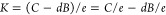	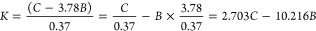 5a	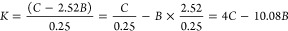 5b
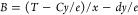	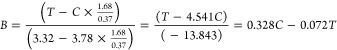 6a	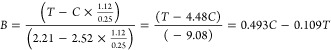 6b

a Refer to Table 1 for definitions of all symbols.

b Refer to Table 2 for the four coefficients determined using the arbitrary units.

c Refer to Table 2 for the four coefficients determined using the units of TId and CId.

d Either the arbitrary or the absolute units, once selected to express CIA and TIA, need to be consistent for purified inhibitors and test samples.

### 3.3 Demonstration and Validation with Mixtures of Purified KTI and BBI

For demonstrating and validating the new methodology to estimate PI composition, purified KTI and BBI were mixed at varying ratios with KTI in the mixtures increasing from 0 to 100% in 20% increments. Conversely, BBI in the mixtures decreased from 100% to 0 in 20% decrements. The six mixtures of solutions were then measured for the *C* and *T* values. Since there were two sets of equations generated due to the use of two different inhibition units (Table 3), for demonstration, two sets of templates were used to estimate various parameters shown in Table 1.

When the measured TIA and CIA (*T* and *C*) values were expressed in arbitrary units, the A set equations in Table 3 had to be used to estimate *K* and *B* values. Table 4 shows a template of using arbitrary units for estimating or calculating various parameters for the six mixtures based on relevant equations and symbols. Specifically, using eqs 4a and 6a in Table 3, *K* and *B* values were first estimated, respectively. Since the mixtures were made from purified KTI and BBI, their inhibitor concentrations were expressed as μg/μg, and the concentration of the total PI (BBI + KTI) was maintained at 1 μg/μg for all mixtures. Results show that the KTI content estimated in a middle column (by using the proposed system of the two linear equations) matched very well with the actual PI composition shown in the left columns. Therefore, the observation validated the new methodology well.

**Table 4:** Estimation of Proteinase Inhibitor Composition and Contribution to Total Inhibition in Six Mixtures of Purified Soybean KTI and Soybean BBI with Increasing % KTI Using Linear Equations with Two Variables and Coefficients Based on the Arbitrary Units (CUI and TUI)^a^

attributes	KTI content in mixtures (actual)	BBI content in mixtures (actual)	CIA measured	TIA measured	KTI (*K*) estimated^b^	BBI (*B*) estimated^b^	KTI + BBI (*K* + *B*)	KTI in mixtures (estimated)	CIA by KTI	CIA by BBI	KTI contribution to total TIA	TIA by KTI	TIA by BBI	KTI contribution to total TIA
unit	%	%	CUI/μg	TUI/μg	μg/μg	μg/μg	μg/μg	%	CUI/μg	CUI/μg	%	TUI/μg	TUI/μg	%
symbol or eq no.^b^			*C*	*T*	eq 4a	eq 6a	eq 7	eq 8	*eK*	*dB*	eq 9	*yK*	*xB*	eq 10
1	0	100	3.77	3.32	0.01	1.00	1.00	0.65	0.00	3.77	0.06	0.01	3.31	0.33
2	20	80	3.09	2.97	0.19	0.80	0.99	19.13	0.07	3.02	2.26	0.32	2.65	10.69
3	40	60	2.40	2.64	0.39	0.60	0.99	39.70	0.15	2.25	6.05	0.66	1.98	24.99
4	60	40	1.72	2.30	0.58	0.40	0.98	59.41	0.22	1.50	12.53	0.98	1.32	42.55
5	80	20	1.06	2.03	0.81	0.20	1.01	80.14	0.30	0.76	28.31	1.36	0.67	67.12
6	100	0	0.37	1.68	1.00	0.00	1.00	100.00	0.37	0.00	100.00	1.68	0.00	100.00

a Refer to Table 2 for measured coefficients based on the arbitrary units of TUI and CUI: *d* = 3.78, *e* = 0.37, *x* = 3.32, and *y* = 1.68.

b For *C*, *T*, and eqs 4a and 6a, refer to Table 3. For all others, refer to Table 1.

According to Table 1, once *K* and *B* (concentrations of KTI and BBI) were determined, CIA by KTI could be easily estimated by multiplying coefficient *e* by *K*, while CIA by BBI could be calculated by multiplying coefficient *d* with *B*. The addition of the two CIA values should be equal to the CIA value measured for each mixture. Consequently, the KTI or BBI contribution to the total CIA (in relative %) could be readily estimated. Using similar approaches, TIA by KTI or BBI and the contribution of the two inhibitors to the total TIA (in relative %) could also be estimated (Table 4).

When the measured inhibitory activities *C* and *T* were expressed in absolute amounts of PI inhibited (TId and CId for trypsin and chymotrypsin inhibitions, respectively), the B set equations in Table 3 had to be used. Table 5 shows a template of using the absolute amounts of PI inhibited for estimating or calculating various parameters for the six mixtures based on relevant equations and symbols. Specifically, *K* and *B* values were first estimated using eqs 4b and 6b in Table 3, respectively. Again, the KTI content estimated in a middle column matched very well with the actual PI composition shown in the left columns, further validating the new methodology. Using similar approaches (except that the four coefficients differed), CIA by KTI or BBI and the contribution of the two inhibitors to the total CIA (in relative %) and TIA by KTI or BBI and the contribution of the two inhibitors to the total TIA (in relative %) could all be estimated (Table 5). Comparison of Tables 4 and 5 shows that using the two sets of coefficients for eqs 1 and 2 (Table 3) gave almost identical values for all parameters estimated, as long as the units of *C* and *T* matched the units of purified inhibitors for coefficient determination (Table 2).

**Table 5:** Estimation of Proteinase Inhibitor Composition and Contribution to Total Inhibition in Six Mixtures of Purified Soybean KTI and Soybean BBI with Increasing % KTI Using Linear Equations with Two Variables and Coefficients Based on the Absolute Units (CId and TId)^a^

attributes	KTI content in mixtures (actual)	BBI content in mixtures (actual)	CIA measured	TIA measured	KTI (*K*) estimated^b^	BBI (*B*) estimated^b^	KTI + BBI (*K* + *B*)	KTI in mixtures (estimated)	CIA by KTI	CIA by BBI	KTI contribution to total CIA	TIA by KTI	TIA by BBI	KTI contribution to total TIA
unit	%	%	μg CId/μg	μg TId/μg	μg/μg	μg/μg	μg/μg	%	μg CId/μg	μg CId/μg	%	μg TId/μg	μg TId/μg	%
symbol or eq no^b^			*C*	*T*	eq 4b	eq 6b	eq 7	eq 8	*eK*	*dB*	eq 9	*yK*	*xB*	eq 10
1	0	100	2.51	2.21	0.01	1.00	1.00	0.97	0.00	2.51	0.10	0.01	2.20	0.49
2	20	80	2.06	1.98	0.19	0.80	0.99	19.43	0.05	2.01	2.34	0.22	1.76	10.89
3	40	60	1.60	1.76	0.40	0.60	0.99	39.94	0.10	1.50	6.19	0.44	1.32	25.21
4	60	40	1.15	1.53	0.58	0.40	0.98	59.20	0.14	1.01	12.59	0.65	0.88	42.38
5	80	20	0.71	1.35	0.81	0.20	1.01	80.02	0.20	0.51	28.43	0.90	0.45	66.99
6	100	0	0.25	1.12	1.00	0.00	1.00	100.00	0.25	0.00	100.00	1.12	0.00	100.00

a Refer to Table 2 for measured coefficients based on the absolute units of TId & CId: *d* = 2.52, *e* = 0.25, *x* = 2.21, and *y* = 1.12.

b For *C*, *T*, and eqs 4b and 6b, refer to Table 3. For all others, refer to Table 1.

### 3.4 Demonstration and Validation with KTI-Null and Wild-Type Soybeans

In a separate experiment (experiment 3), the new methodology for determining PI composition and other parameters was applied to four selected soybean germplasms: two conventional soybean cultivars and two KTI-null ones (Table 6). Among them, the Kunitz cultivar was developed by backcrossing cultivar Kin Du (PI 157440) with Williams 82 (conventional soybeans or wild-type).^30^ Like Kin Du, Kunitz was also known as KTI-null. Besides Williams 82, the other wild-type was an unknown soybean cultivar purchased locally.

**Table 6:** Application of the Proposed Methodology to Two Wild-Type and Two KTI-Null Soybeans for Estimation of Proteinase Inhibitor Composition and Contribution of KTI and BBI To Total Trypsin Inhibition and Chymotrypsin Inhibition^a^

attributes	CIA measured	TIA measured	KTI (*K*) estimated^b^	BBI (*B*) estimated^b^	KTI + BBI (*K* + *B*)	KTI content in mixtures	CIA by KTI	CIA by BBI	BBI to total CIA	TIA by KTI	TIA by BBI	BBI to total TIA
unit	mg CId/g	mg TId/g	mg/g	mg/g	mg/g	%	mg CId/g	mg CId/g	%	mg TId/g	mg TId/g	%
symbol or eq no.^b^	*C*	*T*	eq 4b	eq 6b	eq 7	eq 8	*eK*	*dB*	eq 9	*yK*	*xB*	eq 10
unknown wild type^c^	20.19	32.07	15.95	6.43	22.38	71.26	3.99	16.21	80.26	17.86	14.21	44.31
Williams 82, wild type	18.37	28.59	13.85	5.92	19.77	70.07	3.46	14.91	81.15	15.51	13.08	45.74
Kin Du (PI 157440)^d^	14.89	17.03	4.41	5.47	9.88	44.62	1.10	13.79	92.60	4.94	12.09	71.00
Kunitz (PI 542044)^d^^,^^e^	19.86	21.70	4.76	7.41	12.16	39.09	1.19	18.67	94.01	5.33	16.37	75.46

a For *T*, *C*, and eqs 4b and 6b, refer to Table 3. For all others, refer to Table 1.

b Refer to Table 2 for measured coefficients based on the absolute units of TId & CId: *d* = 2.52, *e* = 0.25, *x* = 2.21, and *y* = 1.12.

c Purchased from a local grocery store.

d Provided by the USDA Soybean Germplasm Collection, Urbana, IL 61801, USA.

e Previously identified as L81-4590 (Bernard et al. 1991).

Upon measurements of CIA and TIA in the samples, it was found that CIA in the two KTI-null samples had an average of 17.38 mg CId/g sample, while CIA in the two conventional cultivars had an average of 19.28 mg CId/g sample. Therefore, the KTI-null beans had only a 10% reduction in CIA over the wild types. In contrast, KTI-null soybeans had significantly lower TIA than conventional soybeans. Specifically, the two KTI-null cultivars had an averaged TIA of 19.38 mg TId/g sample, while the two wild-type beans had an averaged TIA of 30.33 mg TId/g sample. Therefore, TIA in KTI-null cultivars was about 64% of the wild types (or a 36% reduction in TIA) (Table 6). Orf and Hymowitz^31^ first reported that soybeans lacking KTI, including cultivar Kin Du (PI 157440) and others, have 30 to 50% less TIA than that of Amsoy 71, another wild-type cultivar. Liener and Tomlinson^32^ showed that the TIA of untreated PI 157440 soybeans was about 55% TIA and 75% CIA in an untoasted defatted soy flour made from a common variety (that is, 45% TIA and 25% CIA reduction, respectively). The 36% TIA reduction in KTI-null beans observed in the present study was within the range reported by Orf and Hymowitz,^31^ but the 10% CIA reduction observed in the present study was less than that reported by Liener and Tomlinson.^32^ The difference can be attributed to the differences in methods for TIA and CIA measurements and cultivars used as controls (wild type).

When the CIA and TIA values were entered into the system of two linear equations with two variables using B set coefficients (eqs 1b and 2b, Table 3), the concentrations of KTI and BBI in the samples were determined, along with concentrations of total PI and % KTI (Table 6). The four samples had similar BBI concentrations with a range of 5.47–7.41 and an average of 6.31 mg/g sample. However, the two KTI-null cultivars had significantly lower KTI concentrations (an average of 4.58 mg/g) than the two conventional beans (an average of 14.90 mg/g), leading to about 70% reduction in KTI concentrations. Consequently, the total PI content in the KTI-null soybeans was about half that of the wild types. KTI constituted about 70% of total PI in conventional soybeans but was reduced to about 42% in the KTI-null soybeans. Since soybeans contain about 40% proteins based on the total PI content shown in Table 6, KTI and BBI together constituted 5–6% of total seed proteins in conventional soybeans but only about 3% of total seed proteins in KTI-null beans.

Over the past several decades, there have been a few reports comparing KTI-null beans with conventional beans with respect to KTI and/or BBI concentrations. Most used similar cultivars but different methods in measurement and estimation. Brandon et al.^20^ were the first group to measure the BBI content of Williams 82 and an isoline, L81-4590, using an ELISA method they developed and found that the two soybean cultivars had similar CIA as well as BBI content (about 3.0 mg/g). The isoline was shown to lack KTI and was later registered as a Kunitz cultivar.^30^ Further work by the same group^33^ showed that, although the two cultivars had the same BBI content, they differed significantly in the KTI content (7.6 mg/g for Williams 82 vs 0.01 mg/g for the isoline). Therefore, KTI constituted about 71.2% of total PI in Williams 82 but almost 0% in the Kunitz cultivar.

Pesic et al.^21^ analyzed PIs in 12 soybean genotypes consisting of one KTI-null (Lana cultivar) and 11 normal cultivars using native PAGE and scanning densitometry to investigate the varietal effect on PI composition and TIA. They found that, although KTI was undetectable in Lana, the remaining genotypes had KTI concentrations ranging from 4.28 to 6.85% of total extractable protein with the majority around 4.5%. Within the 12 genotypes, the BBI content varied from 0.6 to 6.32% of total extractable proteins, with the majority ranging from 0.97 to 3.30%. The total PI content ranged from 2.32 to 13.17% of total extractable proteins, with most around 5%. Assuming that soybeans contain 40% protein and have a protein extractability of 75%, by a simple conversion (multiplying by 0.4 and then by 0.75), most genotypes would have KTI content around 13.5 mg/g, BBI content from 2.91 to 9.90 mg/g, and total PI around 15 mg/g.

There are also reports on KTI and BBI concentrations in conventional soybeans. Zhou et al.^24^ determined the KTI concentration in the seeds of ten soybean genotypes through two-dimensional chromatography and reported a range of 6.13–8.08 mg/g of defatted flour, with an average value of 6.94 mg/g of defatted flour. They did not measure the BBI content for the study, though. Kumar et al.^22^ measured the contents of KTI and BBI in seven selected soybean genotypes through native PAGE-densitometry and ELISA, respectively. The KTI concentration ranged from 8.70 to 18.53 mg/g of defatted flour, with an average of 12.8 mg/g, while the BBI content ranged from 7.40 to 23.4 mg/g defatted soy flour, averaging 15.54 mg/g. Yang et al.^23^ measured the contents of KTI and BBI in 93 soybean samples from different sources and harvest years by SDS-PAGE and found that KTI contents ranged from 5.25 to 14.60 mg/g sample, and BBI contents ranged from 1.81 to 5.74 mg/g sample.

There are three noticeable variations among studies regarding KTI and BBI contents in raw soybeans. The first one relates to the BBI content relative to the KTI content in normal cultivars. Most previous studies^21,23,33^ and the present study showed that the BBI content is significantly less than the KTI content, but Kumar et al.^22^ reported that the BBI content is as the same as or even higher than the KTI content. The second variation relates to the KTI content in normal cultivars. Some previous studies^21,22^ and the present study showed that the KTI content was >10 mg/g sample, while others^24,33^ reported that the KTI content was <10 mg/g.

The third striking difference among studies relates to the KTI content in KTI-null soybeans. Early reports using either PAGE^21,31^ or ELISA^19,33^ showed undetectable amounts of KTI in KTI-null soybeans. Yet, later reports^10,11,25^ showed that KTI in KTI-null soybeans, which resulted from mutations in the KTi3 gene (e.g., PI 157440, Kunitz cultivar, and PI 547656), the KTi1 gene (e.g., PI 68679), or both genes, was not 100% eliminated. By using the enzymatic and algebraic methodology, the present study (Table 6) shows that the KTI content in KTI-null soybeans was about 1/3 of the KTI content of wild-type soybeans, a 2/3 reduction in KTI instead of the 100% reduction found in the early reports. Therefore, the present study aligns with the later reports.^10,11,25^ The large variation in contents of KTI and BBI of common soybean cultivars and in KTI-null beans among studies can be attributed to the differences in methods of extraction and measurements and in genotypes selected. Advancements in analytical methods and instrumentation are also a contributing factor.

### 3.5 Tricine SDS-PAGE of the Four Soybean Accessions for Further Validation

**Figure 2:**
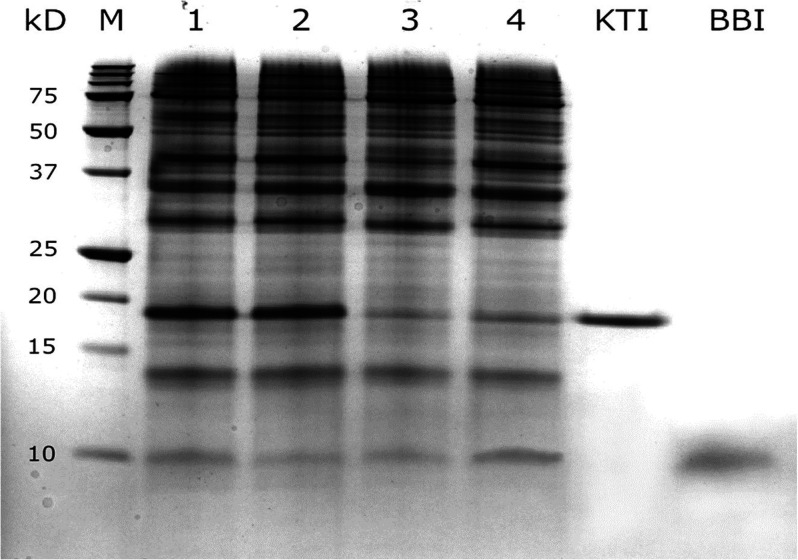
Tricine SDS-PAGE patterns of four soybean accessions, along with purified KTI and purified BBI. Lanes 1–4 represent samples of wild-type 1 (unknown cultivar), wild-type 2 (Williams 82), KTI-null 1 (Kin Du), and KTI-null 2 (Kunitz), respectively. Lane M represents protein markers with descending MWs.

Nonreducing tricine SDS-PAGE was performed for extracts of the four soybean accessions (two KTI-nulls and two wild types) to visually identify any differences in PI composition based on band intensity against the two purified inhibitors (KTI and BBI) and to confirm the finding regarding KTI and BBI contents in these beans (Table 6). As shown in Figure 2, there were many distinct bands, indicating that soy extracts contained many proteins. In the lower half of the gel, there were three distinct bands with low MW. One of them was KTI, with a MW of around 18 kDa. The lowest visible band, corresponding to a MW of about 9 kDa, was BBI. More importantly, the two KTI-null beans still showed a visible KTI band, although it was much weaker than that of the wild types. This indicates that the two KTI-null soybeans still contained some residual KTI. Furthermore, the four soybean accessions showed a similar BBI band intensity, indicating that they had a similar BBI content. Therefore, the SDS-PAGE patterns and intensity of the four soybean accessions (Figure 2) not only provide strong visual evidence to support the KTI and BBI content determined by the EA methodology (Table 6) but also show that the so-called KTI-null soybeans still contain some residual KTI.

The observation that there is still some residual KTI in KTI-null genotypes by previous studies^10,11^ and the present study (Table 6 & Figure 2) is consistent with molecular studies on soybean KTI genes. According to Wang et al.,^11^ the soybean genome contains as many as 38 KTi genes. Among them, three KTi genes (KTi1, KTi2, and KTi3) have been cloned and sequenced.^8^ Further work showed that the low TIA of PI 157440 and its variants (such as the Kunitz cultivar) results from a frameshift mutation in the KTi3 gene.^34^ Another soybean germplasm accession (PI 68679) was found to carry a nonfunctional mutation on the KTi1 gene.^10^ Most recently, a new soybean germplasm accession (IT 105782) was identified by a Korean group,^35^ which has a reduced TIA resulting from an in-frame insertion mutation on the KTi1 gene. Therefore, at least two soybean genes, KTi1 and KTi3, are synergistically controlling the KTI content in soybean seeds.^10,11,35^ The repeated observation that the KTI-null soybeans have substantial TIA (about 50–70% of wild types)^10,11,21,31−33,35^ (Table 6) is explainable by the presence of not only residual KTI but also unaltered BBI, which had inhibition toward trypsin twice as strong as KTI (Table 2).

Finally, several soybean accessions found or bred to have significantly reduced TIA (as compared to that of the wild-type) were described as “KTI-null”, “KTI-free”, “lacking KTI”, or “absence of KTI” by earlier investigators.^8,30−34^ Yet, because these beans have since been found to contain residual KTI, these old but common terms should be interpreted with caution. The new term “KTI-low” soybeans should be used. When referring to a particular KTi gene or allele, such as KTi3 or KTi1, the term “null”, “free”, or “lacking” may still be valid^11,35^ due to a natural or genetic alteration of these genes.

### 3.6 Contribution of KTI and BBI toward Total Trypsin or Chymotrypsin Inhibition

Using relevant equations shown in Table 1, additional parameters for the four soybean samples could be estimated for comparison, including CIA by KTI, CIA by BBI, % KTI contribution to total CIA (or % BBI contribution to total CIA), TIA by KTI, TIA by BBI, and % KTI contribution to total TIA (or % BBI contribution to total TIA) (Table 6). For wild-type soybeans, although KTI constituted about 70% of total PI mass, its contribution to total CIA was only about 20% and to total TIA was only approximately 55%. By contrast, although BBI constituted only about 30% of total PI mass, its contribution to total CIA reached about 80%, 4 times higher than that of KTI. At the same time, the BBI contribution to total TIA was around 45%, only 10% less than the KTI contribution to total TIA. For the two KTI-null soybeans, because KTI constituted only approximately 42% of total PI mass, its contribution to total CIA was reduced to approximately 6.5% and the contribution to total TIA to around 27%. Therefore, the mass composition of KTI and BBI in soybeans did not proportionally transform into their relative contribution to total CIA or TIA. Specifically, compared to KTI, BBI was a major contributor to CIA and a substantial contributor to TIA. This observation was also verified from expt. 2, which used different mixtures of purified KTI and BBI shown in Tables 4 and 5. Furthermore, this new finding can be explained by repeated observations that KTI has a strong inhibition toward trypsin but a weak inhibition toward chymotrypsin, while BBI has approximately equal inhibition toward both enzymes.^1,3^ It is also consistent with the finding shown in Table 2, that is, compared with KTI, BBI had approximately 10 times higher inhibition toward chymotrypsin and 2 times higher inhibition toward trypsin, per mass unit basis.

So far, only a few studies have attempted to determine the inhibitory activities and/or contributions of KTI^22,36^ and BBI^22,37^ toward total TIA in soybeans. None have determined the contribution of the two inhibitors toward total CIA in soybeans. Kumar et al.^22^ first measured KTI and BBI contents in seven selected soybeans by native PAGE-densitometry and ELISA, respectively, and then estimated the TIA of a purified KTI reagent as 2.51 mg TId per mg protein, which they determined by the trypsin-KTI complex assay. They also estimated the TIA of a purified BBI reagent as 0.5 mg of TId per mg protein, which they took directly from a specification provided by Sigma-Aldrich Co. (St. Louis, MO, USA) for the purified BBI reagent purchased. They also measured the total TIA by an enzymatic method. Therefore, the method of Kumar et al.^22^ features measurement of KTI and BBI contents followed by the estimation of the inhibitory activity of individual inhibitors (KTI and BBI) using conversion factors determined by themselves or obtained from a reagent vendor. Unfortunately, the same study showed that the sum of TIA calculated for KTI and BBI was significantly lower than the total TIA determined for all seven genotypes. One genotype even showed less than half of the measured TIA values. The observation indicates that the estimation method of Kumar et al.^22^ can be very unreliable. One major reason for the unreliable estimation is that the actual value in mg TId/mg BBI measured for a particular lot often differs significantly from the specification for BBI provided by a vendor.^28^

The same research group had two follow-up reports. One focused on KTI and its contribution to total TIA.^36^ It showed that among 102 soybean genotypes, the KTI content ranged from 0.1 to 15.9 mg/g sample (averaging 9.4 mg/g sample), while the KTI contribution to total TIA ranged from 1.0 to 79.8% with an average of 52.8%. The other report focused on BBI and its contribution to total TIA.^37^ It showed that among 90 soybean genotypes, the BBI content ranged from 2.2 to 24.5 mg/g sample (averaging 9.3 mg/g sample), while the BBI contribution to total TIA ranged from 2.2 to 53.5% with an average of 11.6%. These two reports led to the conclusion that KTI contributes more than BBI toward total TIA. Yet, this conclusion can be misleading since both reports used the same unreliable method of Kumar et al.^22^ cited above. Other researchers also believe that KTI plays a greater role than BBI toward protease inhibition due to its higher content in raw soybeans.^23,33,35^ The present study used a different strategy to calculate the contribution of individual inhibitors toward total inhibition. It was based on measurements of both TIA and CIA followed by the calculation of inhibitor concentrations by solving the system of linear equations with two variables and coefficients determined with purified inhibitors. Consequently, an opposite conclusion was reached; that is, because KTI contributed significantly less than BBI toward CIA and just slightly higher than BBI toward TIA, it plays a smaller role toward total protease inhibition than BBI.

### 3.7 Further Discussion

For studying PI in soybeans and other legumes, total TIA is often measured, and occasionally CIA is also measured. The methods for assaying inhibitory activities are mostly colorimetric and relatively straightforward, and thus, they have been used ever since soybean KTI was discovered.^3^ Yet, for measuring the content of a particular PI, such as KTI and BBI in soybeans, the methods can be complex, and their development has been relatively recent. During the 1960s and 70s, gel electrophoresis and immunoassays were developed but mostly for qualitative purposes.^31^ Their use for quantitating KTI and BBI in soybeans became possible only after the arrival of reliable imaging technology and associated software.^19,22,29^ Liquid chromatography methods for quantitating inhibitor content arrived just a few years ago.^24−26^ These methods and variants have been used in studies requiring measurement of KTI and/or BBI contents.^11,23,36^ However, many of these methods suffer from one or more inherent problems, including complex procedures, requirements of specific instruments, specific application to KTI or BBI, time- and labor-intensity, cost inefficiency, and the tendency of variable results among studies.

For example, PAGE is a popular technique to detect and characterize proteins, but the accurate migration of proteins on a gel depends on protein characteristics, including amino acid sequence, isoelectric point, structure, and the presence of certain side chains or prosthetic groups. Consequently, some proteins do not migrate according to their MW. Furthermore, quantitation of KTI and BBI by PAGE requires staining, destaining, and densitometric measurements, but each step in the procedure involves several factors, which contribute to difficulties in obtaining reliable quantitative data.^38^ In addition, the PAGE technique can separate proteins by MW but may not differentiate the active and inactive forms of the same inhibitor protein. This may also be true for the HPLC method. Immunoassays are based on antibodies raised against either a native protein (such as KTI or BBI) or its denatured forms. Although the methods can have high sensitivity and high throughput, they suffer from limitations such as antibody availability, high variability, and a limited range of specificities. Furthermore, since proteins undergo structural changes during processing, which in turn affect the antibody–antigen interaction during the immunoassays, choosing or making a wrong type of antibody can lead to erroneous results.^39^ Therefore, many of the reported methods are more suitable for qualitative rather than quantitative measurements of KTI and/or BBI.

The new methodology described in this study offers an alternative way for quantitative estimation of both KTI and BBI concentrations in soybeans and soy products. It features accurate enzymatic measurements of TIA and CIA in a soy sample and subsequent determination of KTI and BBI by solving the system of linear equations with two variables. Therefore, it can be described as an enzymatic and algebraic methodology or simply the EA methodology. As discussed earlier, the new methodology has a theoretical basis for its development. Soybeans contain two types of PIs of protein nature, namely, KTI and BBI. Yet, the two inhibitors show different levels of inhibition toward trypsin and chymotrypsin, two key enzymes in the mammalian digestive tract. KTI has a strong inhibition toward trypsin but a weak inhibition toward chymotrypsin,^3^ while BBI has almost equal inhibitory actions toward both enzymes.^1,6^ It is based on this principle that a system of linear equations with two variables is proposed (Table 1).

Like all other methods, the EA methodology has pros and cons. Its pros include: (1) having a capacity to measure all parameters relating to the two inhibitors in soybeans (total TIA, total CIA, concentrations of KTI and BBI, total PI concentration, % KTI in total PI, % BBI in total PI, TIA by KTI, TIA by BBI, CIA by KTI, CIA by BBI, % contribution of KTI or BBI to total TIA, and % contribution of KTI and BBI to total CIA), (2) requiring no expensive instrumentation, (3) measuring the content of inhibitors that are reactive only as the methodology is based on total TIA and CIA of a test soybean sample, and (4) requiring little effort for method development as the recently improved methods for TIA and CIA assays are readily available. The cons of the EA methodology include: (1) being limited to soybeans or soybean products that are either made 100% from soybeans or contain nonsoy components having no TIA and no CIA, and (2) requiring highly sensitive and highly accurate methods for TIA and CIA assays. In fact, addressing the second con is vital for achieving accurate estimates of all parameters. The recent work at our USDA laboratory for significantly improving the methods for TIA and CIA measurements^16−18,27,28^ has provided a solid basis and made it possible to develop the EA methodology.

In summary, the present study dealt with the development of a new methodology for determining reactive KTI and BBI contents in soybeans as well as other quantitative parameters relating to the two inhibitors. It features enzymatic measurements of TIA and CIA in a test sample, followed by solving a system of linear equations with the two variables proposed in this study. This enzymatic and algebraic methodology was validated by applying it to a series of mixtures of purified KTI and BBI and to soybean seeds of two KTI-null and two wild-type accessions. It serves as a much-needed analytical tool for researchers to achieve various objectives relating to soybean proteinase inhibitors. The new methodology allows the calculation of the contribution of individual inhibitors toward total inhibition with ease. It was first found that although BBI constituted only about 30% of total PI mass in normal raw soybeans, it contributed about 80% toward total CIA (KTI contributes only 20%) and about 45% toward TIA. Furthermore, the study showed that the so-called KTI-null soybean mutants should be called KTI-low soybeans since they still contain measurable KTI. This finding was further confirmed by running SDS-PAGE. Additional work is being conducted to determine if the EA methodology can also be applied to heated soybeans and soy products. The results will be documented in a separate report.

## Abbreviations

BAEE: *N*α-benzoyl-l-arginine ethyl ester
BAPA: *N*α-benzoyl-dl-arginine-*p*-nitroanilide
BBI: Bowman–Birk inhibitor
BTEE: *N*-benzoyl-l-tyrosine ethyl ester
BTNA: *N*-benzoyl-l-tyrosine-*p*-nitroanilide
CIA: chymotrypsin inhibitor activity
CId: chymotrypsin inhibited
CUI: chymotrypsin units inhibited
ELISA: enzyme-linked immunosorbent assay
KTI: Kunitz trypsin inhibitor
PAGE: polyacrylamide gel electrophoresis
PI: protease inhibitors
SDS: sodium dodecyl sulfate
TIA: trypsin inhibitor activity
TId: trypsin inhibited
TUI: trypsin units inhibited
